# Metabolism and Bioactivation of Corynoline With Characterization of the Glutathione/Cysteine Conjugate and Evaluation of Its Hepatotoxicity in Mice

**DOI:** 10.3389/fphar.2018.01264

**Published:** 2018-11-06

**Authors:** Ruijuan Liu, Fang Zhou, He He, Jingyao Wei, Xin Tian, Li Ding

**Affiliations:** ^1^Department of Pharmacy, The First Affiliated Hospital of Zhengzhou University, Zhengzhou, China; ^2^Department of Pharmaceutical Analysis, China Pharmaceutical University, Nanjing, China; ^3^Department of Pharmacy, The First Affiliated Hospital of Xinxiang Medical University, Xinxiang, China; ^4^Department of Physiology and Pharmacology, China Pharmaceutical University, Nanjing, China

**Keywords:** corynoline, metabolism, bioactivation, CYP450 enzymes, hepatotoxicity, mass spectrometry

## Abstract

Corynoline (CRL), an isoquinoline alkaloid, is the major constituent derived from *Corydalis bungeana* Herba, which is a well-known Chinese herbal medicine widely used in many prescriptions. The purpose of this study was to comprehensively investigate the metabolism and bioactivation of CRL, and identify the CYP450 isoforms involved in reactive *ortho*-benzoquinone metabolites formation and evaluate its hepatotoxicity in mice. Here, high resolution and triple quadrupole mass spectrometry were used for studying the metabolism of CRL. Three metabolites (M1–M3) and four glutathione conjugates (M4–M7) of CRL *ortho*-benzoquinone reactive metabolite were found *in vitro* using rat and human liver microsomes supplemented with NADPH and glutathione. Four cysteine conjugates (M8–M11) were trapped in mice besides M1–M7. Using human recombinant CYP450 enzymes and chemical inhibitor method, we found that CYP3A4, CYP2C19, CYP2C9, and CYP2D6 were mainly involved in the bioactivation of CRL. Furthermore, CRL had no obvious hepatotoxicity and did not induce acute liver injuries in the experimental dosage (125–500 mg/kg) used in this study. However, phenomena of abnormal behaviors and low body temperature appeared in mice after drug administration, and three of them were dead. Tissue distribution study of CRL in mice showed that the main target organ of CRL was liver, then kidney, heart, and brain. CRL could traverse the blood–brain barrier, and have relative high concentration in brain. So, we surmise that toxicity effect of CRL on other organs may have occurred, and more attention should be paid on the traditional Chinese medicine contained CRL in clinic.

## Introduction

**FIGURE 1:**
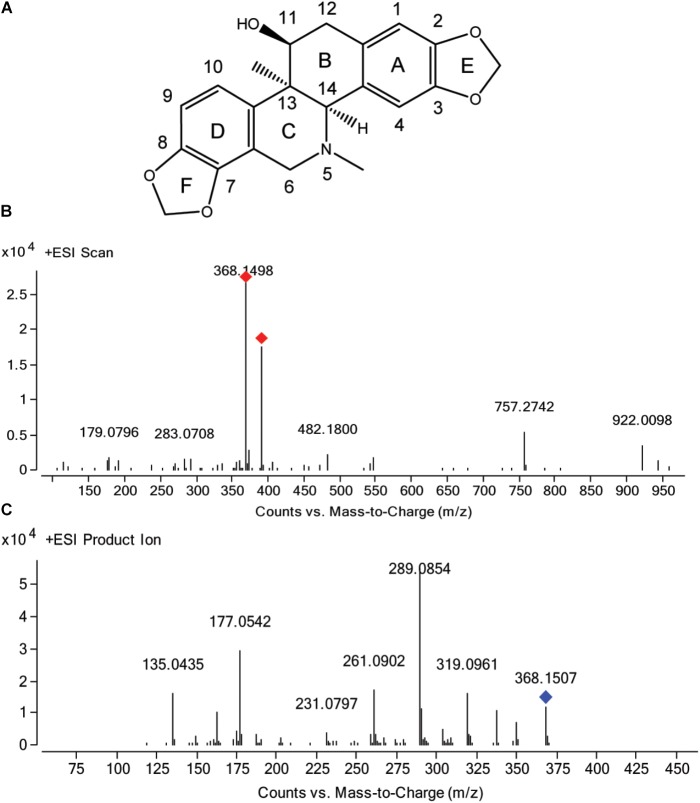
Chemical structure of corynoline **(A)** and the mass spectra of corynoline in full scan **(B)** and product ion scan **(C)** with the precursor ion of [M+H]^+^.

Corynoline (CRL, Figure 1), an isoquinoline alkaloids, is the major active constituent derived from *Corydalis bungeana* Herba (Kim et al., 2000; Niu et al., 2011). *Corydalis bungeana* Herba is a well-known traditional Chinese herb recorded in the Chinese Pharmacopoeia Commission (2015), and widely used in many traditional Chinese Medicine prescriptions, such as Shuanghua Baihe tablets and Ganmao Qingre Granule (Liu et al., 2015, 2016). CRL is the only ingredient for assessing the quality of this herbal medicine. Furthermore, it has been proved that CRL have many pharmacology activities, including anti-inflammation (Dong et al., 2015; Zhai et al., 2016), cell adhesion inhibitory (Kamigauchi et al., 2005), inhibition of acetylcholinesterase (Kim, 2002) and β-site amyloid precursor protein cleaving enzyme 1 (BACE1) (Chlebek et al., 2016), cytotoxic activities (Choi et al., 2007) and so on, which have captured the interest of scientists in recent years.

With the traditional Chinese medicines having been used increasingly and extensively in worldwide recently, security of Chinese herb medicine becomes more and more significant and has attracted the attention of researchers (Li et al., 2016; Ng et al., 2017; Khojasteh et al., 2018). Is there any relationship between metabolism and drug toxicity? Mostly, the biological activity of parent drug can loss after the hepatic metabolism mediated by cytochrome P450 enzymes (CYP450, Phase I reactions and Phase II conjugations), and such metabolic reactions are regarded as detoxification pathways. However, depending on the structural features present in some xenobiotics, the same metabolic events may generate chemically reactive and toxic metabolite occasionally (Kalgutkar and Soglia, 2005; Amaya et al., 2018). According to the previous studies, researchers had reviewed some functional groups commonly contained in drug or xenobiotics, and the biotransformation of relatively inert organic compounds to reactive metabolites (reactive electrophilic intermediates), which is commonly referred to as metabolic activation or bioactivation, has been speculated to contribute toward certain drug-induced toxicities, including hepatotoxicity and others (Kalgutkar and Soglia, 2005; Schmidt et al., 2013; Teschke and Eickhoff, 2015). Reactive metabolites, if not detoxified, could covalently modify essential cellular targets, including protein and DNA, which would result in toxicological response (Chan et al., 2018; Khojasteh et al., 2018).

The methylenedioxyphenyl, contained in the chemical structure of CRL, was an alert group, which may occur metabolic activation *in vivo* (Murray, 2000). Therefore, it is necessary to understand the metabolic pathway and evaluate the potential toxicity of CRL. Fang et al. (2011) identified the CYP450 isoforms involved in the metabolism of CRL, and two metabolites were found which were catalyzed by CYP2C9 and CYP3A4. Mao et al. (2015) characterized the reactive intermediates of CRL by detecting the N-acetylcysteine conjugation. In our previous study (Liu et al., 2015), we investigated the pharmacokinetics and absolute bioavailability of CRL in rats. Choi et al. (2007) reported that CRL exhibited cytotoxicity against human A549, SK-OV-3, SK-MEL-2, and HCT15 tumor cells, and the 50% growth inhibition values were 5.27–6.14 μM. To date, as far as we know the more detail information on metabolism and hepatotoxicity of CRL in mice has not been reported. Although not all reactive metabolites can cause hepatotoxicity and not all covalent binding events lead to a deleterious biological consequence, inadequate detoxification of reactive metabolites is a risk factor for liver or other organs injury. Therefore, more research needs to be conducted for gain insight into the property of CRL and better apply to the diseases treatment.

The purpose of this study was to (1) comprehensively investigate the metabolism and bioactivation of CRL using reduced glutathione (GSH) as a trapping agent and (2) identify the CYP450 isoforms involved in reactive *ortho*-benzoquinone metabolites formation and (3) evaluate its hepatotoxicity in mice. This study provided evidence for the reasonable application and further research on the traditional Chinese medicine contained CRL in clinic.

## Materials and Methods

### Chemicals and Reagents

The reference standard of CRL (purity 100%) was purchased from the National Institutes for Food and Drug Control (Beijing, China). CRL (purity >98%) for incubation in liver microsomes and dosage administration were purchased from Shanghai Tauto Biotech Co., Ltd. (Shanghai, China). Reduced L-glutathione (GSH), β-nicotinamide adenine dinucleotide 2′-phosphate reduced tetrasodium salt (NADPH), thiotepa (CYP2B6 inhibitor), quercetin (CYP2C8 inhibitor), ticlopidine hydrochloride (CYP2C19 inhibitor) and 4-methylpyrazole (CYP2E1 inhibitor) were purchased from Sigma-Aldrich (St. Louis, MO, United States). Alpha-naphthoflavone (CYP1A2 inhibitor), quinidine (CYP2D6 inhibitor), and ketoconazole (CYP3A4/5 inhibitor) were purchased from Tokyo Chemical Industry (Shanghai) Co., Ltd. (Shanghai, China). Sulfaphenazole (CYP2C9 inhibitor) was purchased from Dr. Ehrenstorfer GmbH (Augsburg, Germany). Human liver microsomes (50 donors) and recombinant human CYP isoforms (CYP1A2, CYP2B6, CYP2C8, CYP2C9, CYP 2C19, CYP2D6, CYP2E1, CYP3A4, and CYP3A5) were purchased from Celsis *In Vitro* Technologies (Baltimore, MD, United States). Methanol and acetonitrile were HPLC grade and purchased from Merck (Darmstadt, Germany). Acetic acid (analytical grade) was purchased from Nanjing Chemical Reagents Co., Ltd. (Nanjing, China). Deionized water was purified using a Milli-Q system (Millipore, Milford, MA, United States). Alanine aminotransferase (ALT) assay kit and aspartate aminotransferase (AST) assay kit were purchased from Nanjing Jiancheng Bioengineering Institute (Nanjing, China).

### Animals

Sprague-Dawley rats were obtained from Shanghai B&K Laboratory Animals Co., Ltd. (Shanghai, China). Ten male rats (200 ± 10 g) were kept in an environmentally controlled breeding room for 1 week before starting the experiments and fed with standard laboratory food and water. Besides, 4-week-old BALB/c male mice of clean grade were bought from the same animal center. Forty mice were acclimated for 1 week before the experiment. Mice were randomly divided into four groups (10 animals per group) and were fasted overnight with free access to water before administration. The animal studies were performed in accordance with the Guide for the Care National Institutes of Health. Ethical approvals for the animal experiments were obtained from the Animal Ethics Committee of China Pharmaceutical University (No. AECCPU201610042).

### Instrumentation and Conditions

#### High Resolution Mass Spectrometry

Two kinds of mass spectrometry were used in this study. One was Agilent 1260 LC system coupled with an Agilent 6540 quadrupole/time of flight mass spectrometry (LC-Q-TOF/MS, Agilent Technologies, Palo Alto, CA, United States). Chromatographic separation was carried on a Hedera ODS-2 C18 analytical column (150 mm × 2.1 mm, 5 μm; Hanbon Science and Technology, Huai’an, China) with a security Guard-C18 column (4 mm × 2.0 mm, 5 μm; Phenomenex, Torrance, CA, United States). The flow rate was 0.3 mL/min and the mobile phase of methanol (solvent A) and water containing 0.1% acetic acid (solvent B) in a linearly gradient program was conducted. The gradient program was as the following condition: 0–3 min, 95% B; 3–15 min, 95–30% B; 15–20 min, 30–10% B; 20–25 min, 10% B; 25–26 min, 10–95% B; 26–32 min, 95% B. The column temperature was maintained at 55°C. The injection volume was 10 μL. The mass spectrometry was equipped with an electrospray ionization source and operated in the positive mode with the full scan range of m/z 100–m/z 1300. Besides, auto MS/MS and targeted MS/MS modes were chosen for obtaining more fragmented ion information of analytes. Drying gas temperature of 350°C with N_2_ gas flow at 10 L/min, nebulizer pressure of 45 psi, capillary voltage of 4000 V, fragmentor of 135 V and collision energy of 25 eV were set.

#### Triple Quadrupole Mass Spectrometry

The other instrument was an Agilent 1200 Series LC (Agilent Technologies, Palo Alto, CA, United States) coupled to an Agilent 6410B triple quadrupole mass spectrometer (United States) equipped with an ESI source. The chromatographic condition was the same as mentioned above except the injection volume of 5 μL and column temperature of 38°C. The data were obtained in positive and negative mode, respectively. Scan range of m/z 150–m/z 900 was set for neutral loss scan. The mass spectrometer was operated with the drying gas temperature of 350°C with N_2_ gas flow at 10 L/min, nebulizer pressure of 50 psi, capillary voltage of 4000 V, fragmentor of 150 V and collision energy of 35 eV. The signal acquisition and peak integration were performed using the MassHunter Qualitative Analysis Software (B.03.01) supplied by Agilent Technologies.

### Preparation of Rat Live Microsome

The male Sprague-Dawley rats were sacrificed by decapitation after a 16 h fasting with free access to water. Liver microsomes were prepared by the refrigerated ultracentrifugation method (Zeng, 2008). Liver samples were excised, rinsed with ice-cold 100 mM phosphate buffered saline (PBS, pH = 7.4), absorbed the solution with filter papers, and being weighed. Add appropriate volume of PBS to the liver sample at ration of 1:3 (weight to volume, g/mL), and ground with glass homogenizers. Then the liver homogenate was centrifuged at 4°C, 9,000 *g* for 25 min, and supernatant was separated followed by centrifuging at 4°C, 105,000 *g* for 60 min. After that pellets were re-suspended with 100 mM PBS containing 20% glycerol and stored t at −80°C immediately. All manipulations were carried out in an ice-cold bath. The concentration of microsomal protein was determined by Beyotime BCA assay kit according to the manufacturer’s instructions (Jiangsu, China). Besides, the enzymatic activity of the rat liver microsome was evaluated according to the previous reported method of our lab (Sun et al., 2013).

### Incubations of CRL in Mouse and Human Liver Microsomes With GSH

Stock solution of CRL was prepared in dimethyl sulfoxide (DMSO). A total of 50 μM CRL was mixed with 1 mg/mL rat or human liver microsomes, 3 mM GSH and PBS (100 mM, pH 7.4) in a final volume of 200 μL. After preincubated at 37°C for 5 min, the mixture were then initiated by adding NADPH (final concentration of 1 mM) and incubated at 37°C for 1 h. The final concentration of DMSO in the incubation is less than 0.1%. Control samples with no NADPH, no GSH, no liver microsomes, or no CRL were also prepared, respectively. The reaction was terminated by adding three volumes of ice-cold acetonitrile. After being vortexed for 5 min, the mixture was centrifuged at 17,000 g for 10 min to precipitate the proteins. The supernatant was collected and evaporated to dryness under a stream of nitrogen at 40°C, and then residue was reconstituted in 100 μL of methanol-water solution (1:1, v/v). A 10 μL aliquot of the reconstituted solution was injected into the LC-Q-TOF/MS or LC-MS/MS system for analysis.

### Cytochrome P450 Involved *in vitro* Metabolism of CRL

#### CYP450 Inhibition by Chemical Inhibitors

Different CYP450-specific inhibitors were used for investigating the specific CYP450 enzyme(s) primarily responsible for the formation of CRL metabolites. Inhibitors and their final concentration for CYP450s enzyme identification were listed in Table 1. The incubation mixtures consisted of CRL (50 μM), HLM (1 mg/mL), GSH (3 mM), individual chemical inhibitors (various concentrations) and PBS (100 mM, pH 7.4) in a final volume of 200 μL. Control samples with no chemical inhibitor were also prepared. Incubations were started by adding NADPH (final concentration of 1 mM), and terminated by adding three volumes of ice-cold acetonitrile containing 20 nM donepezil as an internal standard (IS) after 1 h. The incubation samples were then prepared as the process described above. Each incubation was performed in triplicate. The metabolism and GSH conjugates was monitored and quantitated by LC-MS/MS.

**Table 1:** Inhibitors and their final concentration for CYP450s enzyme identification.

CYP450 isoforms	Inhibitors	Final concentration (μM)
CYP1A2	α-Naphthoflavone	1
CYP2B6	Thiotepa	50
CYP2C8	Quercetin	20
CYP2C9	Sulfaphenazole	10
CYP2C19	Ticlopidine	20
CYP2D6	Quinidine	5
CYP3A4/5	Ketoconazole	1
CYP2E1	4-Methylpyrazole	50

#### Recombinant Human CYP450 Phenotyping

The incubation was performed similarly to those of the microsomal incubations, except that the microsomes were substituted with recombinant cDNA-expressed CYP1A2, CYP2B6, CYP2C8, CYP2C9, CYP2C19, CYP2D6, CYP3A4, CYP3A5, and CYP2E1, respectively. The final concentrations of the CYP450 enzymes were 50 nM. Each incubation was performed in triplicate. Control samples with no NADPH, and no CRL were also prepared, respectively. A total normalized rate (TNR) method (Rodrigues, 1999) was applied to determine the involvement of CYP450(s) in the bioactivation of CRL. The rates of metabolite formation in individual incubations with recombinant CYP450 enzymes (rCYPn) were multiplied by the mean specific content of the corresponding CYP450 enzyme in HLM (mCYPn) to obtain the normalized rate (NR) for each enzyme. TNR was obtained by summing the NR of each CYP450 isoform. The contribution rate of each CYP450 isoform could be calculated by the following formula: Contribution (%) = (NR/TNR) × 100% = (pmol/min/pmol rCYPn × pmol mCYPn/mg)/Σ(pmol/min/pmol rCYPn × pmol mCYPn/mg) × 100%.

### Animal Study and Sample Treatment

#### Hepatotoxicity Study

Thirty-six BALB/c male mice were randomly divided into four groups (nine animals per group) including control group, low (125 mg/kg), middle (250 mg/kg), and high (500 mg/kg) three dosages group. CRL was dissolved firstly in DMSO and then prepared in 0.5% carboxymethyl cellulose sodium (CMC-Na) to make a suspension of 10, 20, and 30 mg/mL and the final concentration of DMSO in the suspension was no more than 5%. CRL was administered to the mice in different groups through oral gavage. Mice in control group were given equal volume 0.5% CMC-Na (contained 5% DMSO) solution with high dosage group. Blood samples were drawn from the orbital vein using cannulation under anesthesia at 24 h after oral administration. After centrifugation at 2,000 *g* for 15 min, the serum samples were stored at −20°C until analysis. Then mice were euthanized by decapitation after blood collection. Samples of liver were obtained and one part of which was fixed in 10% formaldehyde for observing histological change with optical microscope. The content of ALT and AST in mice serum was detected according the operation instruction of the kit.

#### Tissue Distribution of CRL in Mice

Thirty BALB/c male mice were fasted overnight but allowed access to water before drug administration. Five groups of mice (six mice per group) were intragastric administered a single dose of 250 mg/kg CRL. The mice in five groups were euthanized by decapitation at pre-dose, 0.5, 2, 4, and 8 h after dosing, respectively. Tissues including heart, liver, spleen, lung, kidney, and brain were dissected and washed with saline, then were blotted with filter paper and weighed accurately. The tissue samples were homogenized in ice-cold saline (1:3, w/v, g/mL). These tissue homogenates were then stored at −20°C and allowed to thaw at room temperature and vortex-mixed before processing. A 200 μL tissue sample was mixed with 5 μL IS solution (donepezil, 500 ng/mL). The mixture was deproteinized with 800 μL of acetonitrile, vortexed for 5 min, and then centrifuged at 17,000 × *g* for 10 min. An aliquot of 5 μL supernatant was then used for quantifying the content of CRL in each tissue by the LC-MS/MS.

### Characterization and Semi-quantification of CRL Metabolites in Liver

Mouse liver samples were deproteinized with acetonitrile according to the method mentioned above, and then the supernatant was separated and evaporated to dryness under a gentle stream of nitrogen in a water bath of 37°C. The residues were reconstituted with 100 μL methanol-water (1:1, v/v) solution and centrifuged at 17,000 × *g* for 3 min. An aliquot of 10 μL supernatant was then used for the mass spectrometry analysis. The metabolites of CRL in mouse liver were characterized by LC-Q-TOF/MS with the same method used in RLM incubation samples and then were semi-quantified by LC-MS/MS with MRM mode. The chromatographic separation was performed with the aforementioned condition in the item of Section “Instrumentation and Conditions.” The mass spectrometer was operated in the positive ESI mode with the drying gas temperature of 350°C with N_2_ gas flow at 10 L/min, nebulizer pressure of 40 psi, and capillary voltage of 4000 V. The specific other mass parameters for each analyte were displayed in Table 2.

**Table 2:** Multiple reaction monitoring transitions and correlative optimized parameters used for the detection by LC-MS/MS.

Analytes	Transition	Fragmentor (V)	Collision energy (eV)
Corynoline	368.2→289.2	130	25
M1/M2	356.1→277.1	135	25
M3	344.1→277.0	135	25
M4/M5/M6	661.2→337.2	180	35
M7	649.2→376.1	150	45
M8/M9/M10	475.1→337.1	140	35
M11	463.2→376.2	130	25
Donepezil (IS)	380.2→91.1	135	45

### Statistical Analysis

Data were expressed as the mean ± standard deviation (SD) and shown by the bars in the graphs. Group means were compared by one-way analysis of variance (ANOVA) followed by Tukey’s *post hoc* test. Statistical significance is considered at *P* < 0.05.

## Results

### Characteristic Fragment Ions Analysis of CRL by LC-Q/TOF-MS

**FIGURE 2:**
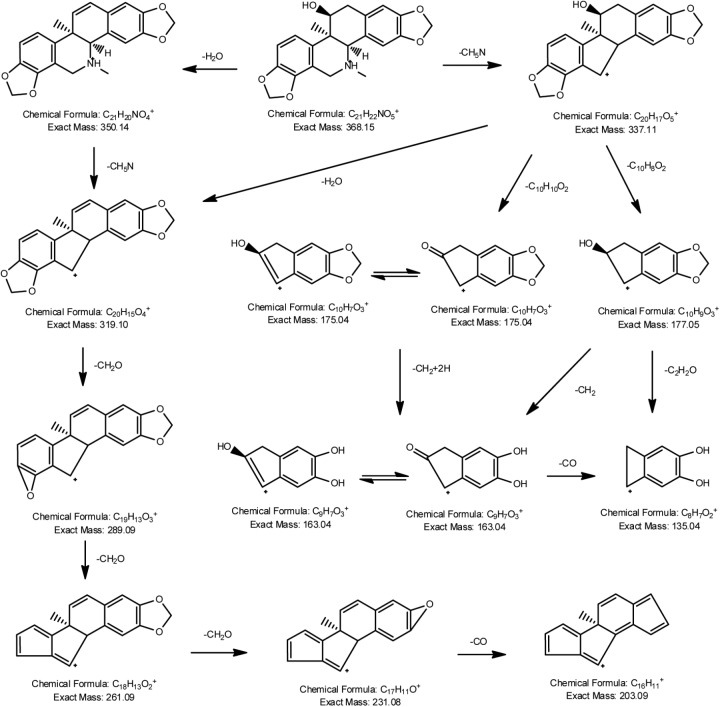
Proposed fragmentation pathways of corynoline.

Auto MS/MS and targeted MS/MS scan modes of Q/TOF were performed in this study. The mass spectra of CRL in full scan and product ion scan with the precursor ion of [M+H]^+^ were shown in Figures 1B,C. Information of the [M+H]^+^ of CRL and its product ions in mass spectrum was listed in Table 3. Molecular ions [M+H]^+^ (m/z 368) and [M+Na]^+^ (m/z 390) were detected in full scan mode. Rings in skeletal structure of CRL were named as A, B, C, D, E, and F, respectively (see Figure 1). Fragment ion m/z 350 (C_21_H_20_NO_4_) was obtained by losing of H_2_O from B ring of [M+H]^+^, which was [M+H-H_2_O]^+^; m/z 337 (C_20_H_17_O_5_) was formed by losing -NH_2_CH_3_ group from the C ring of [M+H]^+^, which was [M+H-NH_2_CH_3_]^+^; m/z 319 (C_20_H_15_O_4_) was derived from a loss of H_2_O and -NH_2_CH_3_ group from B and C ring of [M+H]^+^, respectively, and that was [M+H-H_2_O-NH_2_CH_3_]^+^; m/z 289 (C_19_H_13_O_3_) was formed by losing -CH_2_O group from the F ring of m/z 319, which was [M+H-H_2_O-NH_2_CH_3_-CH_2_O]^+^; m/z 261 (C_18_H_13_O_2_) was formed by further losing -CO group from the F ring of m/z 289, which was [M+H-H_2_O-NH_2_CH_3_-CH_2_O-CO]^+^; m/z 231 (C_17_H_11_O) was formed by losing -CH2O group from the E ring of m/z 261, which was [M+H-H_2_O-NH_2_CH_3_-2CH_2_O-CO]^+^; m/z 177 (C_10_H_9_O_3_) was formed by the cleavage of B ring and further losing -C_10_H_8_O_2_ group from m/z 337, which was [M+H-H_2_O-NH_2_CH_3_-C_10_H_8_O_2_]^+^; m/z 135 (C_8_H_7_O_2_) was formed by losing -C_2_H_2_O group from the B ring of m/z 177, which was [M+H-H_2_O-NH_2_CH_3_-C_10_H_8_O_2_-C_2_H_2_O]^+^. Proposed fragmentation pathway of CRL was exhibited in Figure 2.

**Table 3:** Information of the [M+H]^+^ of corynoline and its product ions in mass spectrum of product ion mode.

Observed m/z	Formula	Calculated m/z	Diff (ppm)	Loss formula
368.1507	C_21_H_21_NO_5_	368.1492	−3.91	–
350.1382	C_21_H_19_NO_4_	350.1387	1.49	H_2_O
337.1065	C_20_H_17_O_5_	337.1071	1.65	CH_4_N
319.0961	C_20_H_15_O_4_	319.0965	1.25	CH_7_NO
289.0854	C_19_H_13_O_3_	289.0859	1.83	C_2_H_8_NO_2_
261.0902	C_18_H_13_O_2_	261.0910	2.91	C_3_H_8_NO_3_
231.0797	C_17_H_11_O	231.0804	3.40	C_4_H_10_NO_4_
203.0846	C_16_H_11_	203.0855	4.56	C_4_H_10_NO_5_
187.0756	C_12_H_11_O_2_	187.0754	2.58	C_9_H_10_NO_3_
177.0542	C_10_H_9_O_3_	177.0546	5.48	C_11_H_12_NO_2_
175.0744	C_11_H_11_O_2_	175.0754	4.93	C_10_H_10_NO_3_
163.0382	C_9_H_7_O_3_	163.0390	4.73	C_12_H_14_NO_2_
135.0435	C_8_H_7_O_2_	135.0441	4.12	C_13_H_14_NO_3_

### Quantitation of CRL Metabolites and Their GSH Conjugates in Liver Microsomes

**FIGURE 3:**
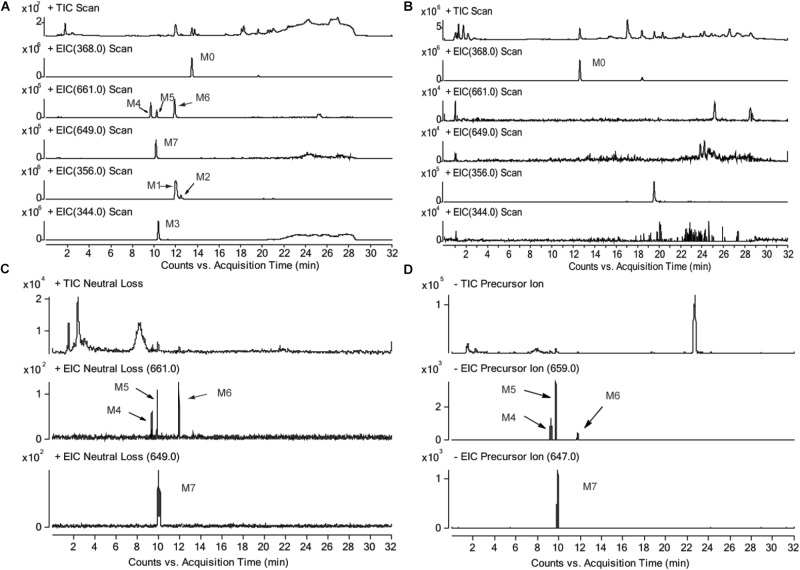
Total ion chromatograms and extracted ion [M+H]^+^ chromatograms of corynoline (M0), metabolites (M1, M2, M3) and their GSH conjugates (M4, M5, M6, M7) in rat liver microsome incubations, **(A)** with NADPH and GSH, **(B)** without NADPH; total ions chromatogram and extracted ion [M+H]^+^ chromatogram of M4-M7 in rat liver microsome incubated with corynoline supplemented with NADPH and GSH, obtained by LC-MS/MS in neural loss mode **(C)** and precursor ion mode **(D)**.

The chromatograms of RLM incubation samples were obtained by using LC-MS/MS in positive and negative mode, respectively, in order to characterizing the metabolite of CRL and their GSH conjugates. Total ion chromatograms and extracted ion [M+H]^+^ chromatograms of CRL (M0), metabolites (M1, M2, and M3) and their GSH conjugates (M4, M5, M6, and M7) in rat liver microsome incubations were shown in Figures 3A,B. Total ions chromatogram and extracted ion [M+H]^+^ chromatogram of M4–M7 in rat liver microsomes incubated with CRL supplemented with NADPH and GSH, obtained by LC-MS/MS in neural loss mode and precursor ion mode were shown in Figures 3C,D.

Three metabolites (M1, M2, and M3) and four metabolite-GSH conjugates were identified in the rat liver microsomes in this study. The chromatographic retention time (RT) of the parent drug (CRL, M0, m/z 368, [M+H]^+^) was 13.6 min. The RTs were 12.0 and 12.5 min for the metabolite M1 and M2, respectively, and molecular ion peak [M+H]^+^ of which both were m/z 356. The RT of the metabolite M3 (m/z 344, [M+H]^+^) was 10.4 min. The RTs of the metabolites M4, M5, M6 (m/z 661, [M+H]^+^) were 9.7, 10.2, and 12.1 min, respectively. The RT of the metabolite M7 (m/z 649, [M+H]^+^) was 10.0 min. No related metabolites were found in the control group without NADPH, which demonstrated that formation of the metabolites was dependent on NADPH or that whether the enzyme reaction was initiated. Then product ion information in mass spectrum of the metabolites (Supplementary Tables 1–3) were analyzed and the possible fragmentation pathways were proposed (Supplementary Figures 1–3).

**FIGURE 4:**
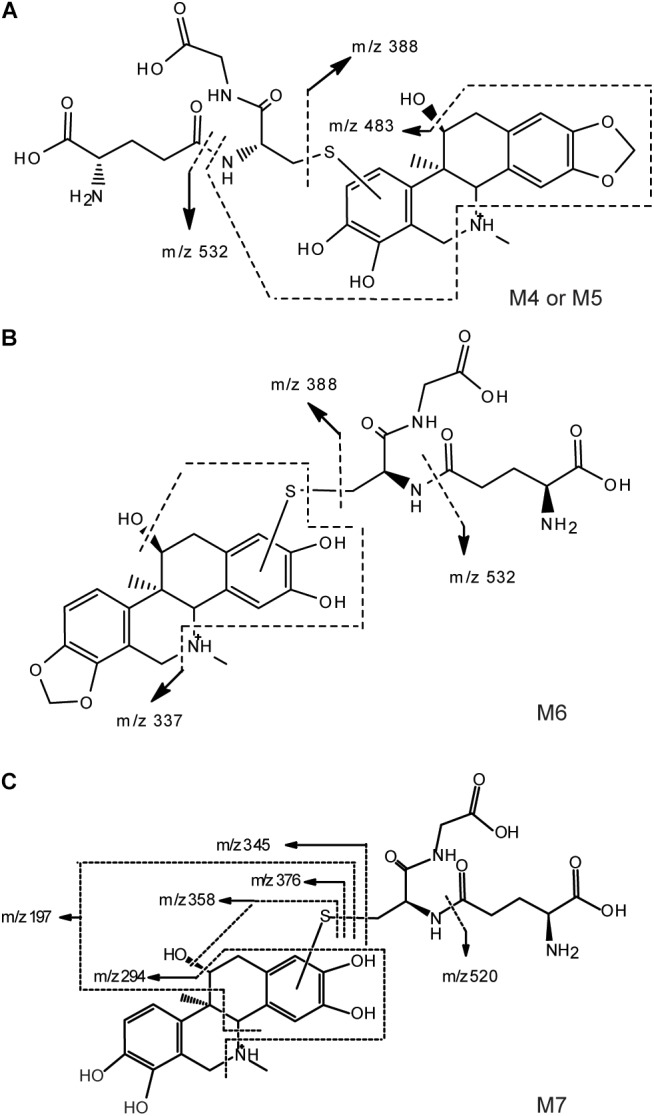
Proposed structure and fragmentation pathways of M4, M5 **(A)**, M6 **(B)** and M7 **(C)**.

[M+H]^+^ of M4–M6 were m/z 661, which was more 305 Da than m/z 356. Combining with the results of neutral loss scan (129 Da) and precursor ion scan (272 Da), we speculate that M4–M6 was formed by conjugating one GSH with M1 and M2, respectively (Figures 4A,B). Then the structures were further verified by analyzing their product ion mass spectrum and the information of the fragment ions (see Supplementary Figures 4, 5). The ions m/z 532 and m/z 388 were formed by losing the pyroglutamic acid (129 Da) and GSH-H_2_S (273 Da), respectively, from M4–M6; m/z 483 was derived from m/z 532 by losing H_2_O from the B ring and losing -NH_2_CH_3_ group from C ring; m/z 337 was derived from m/z 388 by losing H_2_O from the B ring and losing -NH_2_CH_3_ group from C ring. Besides, m/z 177 were found in M4 and M5, but not in M6, which indicated that the E ring was intact and M4, M5 were two isomers formed by one GSH combing with M1 at the 9th or 10th position of the D ring. M6 was formed by combing with one GSH at the A ring of M2.

With the same identification method, [M+H]^+^ of M7 were m/z 649 (see Supplementary Figure 6), which was more 305 Da than m/z 344. m/z 520 and m/z 376 were formed by losing the pyroglutamic acid (129 Da) and GSH-H_2_S (273 Da), respectively, from M7; m/z 358 was derived from m/z 376 by losing H_2_O from the B ring; m/z 345 was derived from m/z 376 by losing -NH_2_CH_3_ group from C ring; m/z 197 was more 32 Da than the fragment ion m/z 165 of M3, which indicated that M7 was the product of M3 combing with one GSH at the A ring (Figure 4C).

Furthermore, the same metabolites of CRL were found in human liver microsome incubation samples as in rat liver mirosomes. Total ion chromatograms and extracted ion [M+H]^+^ chromatograms of CRL (M0), metabolites (M1, M2, and M3) and theirs GSH conjugates (M4, M5, M6, and M7) in human liver microsome incubations were exhibited in Supplementary Figure 7.

### CYP Isoforms Involved in the Formation of Metabolite-GSH Conjugates

**FIGURE 5:**
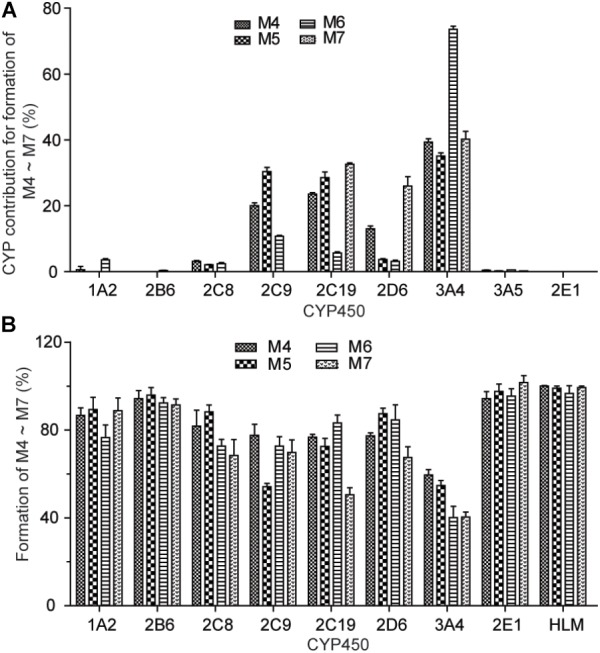
Formation of M4∼M7 with recombinant human CYP450 enzymes **(A)**. Effect of selective CYP450 inhibitors on the formation of GSH conjunction M4–M7 in incubations of corynoline with human liver mirosome **(B)**.

To identify the isoforms of CYP450 enzymes preferentially responsible for the oxidative metabolism of catechol to the *ortho*-benzoquinone intermediate, the formation of the GSH conjugate M4–M6 were investigated in different human recombinant CYP450 enzymes and by the chemical inhibitor method in HLM. The results of the two methods were shown in Figure 5. Contribution rate of each CYP450 isozyme to the production of reactive metabolites M4–M6 was calculated (Supplementary Table 4). The results showed that multiple CYP450 enzyme isoforms mediate the production of reactive metabolites of CRL. Formation of M4 was mainly mediated by CYP3A4, CYP2C19, and CYP2C9, and the contribution rate of which were 39.4, 23.6, and 20.1%, respectively. Formation of M5 was mainly mediated by CYP3A4, CYP2C9, and CYP2C19, and the contribution rate of which were 35.1, 30.4, and 28.5%, respectively. Formation of M6 was mainly mediated by CYP3A4, and the contribution rate of which was 73.5%. Formation of M7 was mainly mediated by CYP3A4, CYP2C19, and CYP2D6, and the contribution rate of which were 40.2, 32.7, and 26.0%, respectively. Above knowable, CYP3A4, CYP2C19, CYP2C9, and CYP2D6 were mainly involved in the bioactivation of CRL.

### Hepatotoxicity Evaluation of CRL in Mice

**FIGURE 6:**
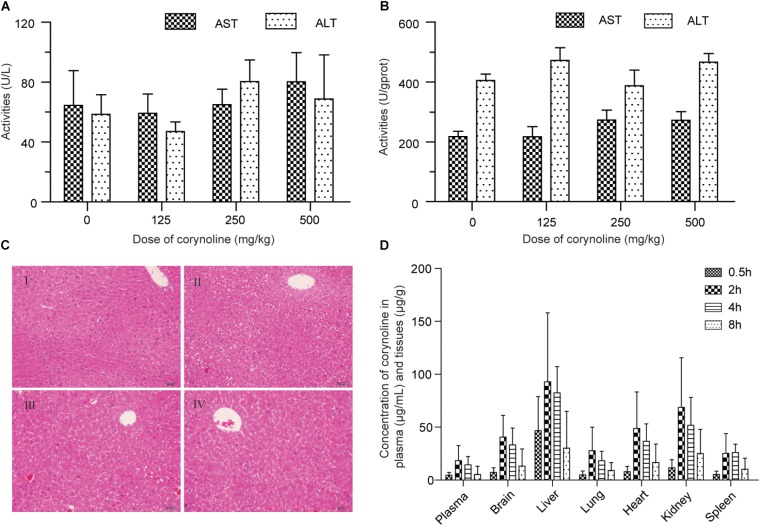
Effects of corynoline on the ALT and AST in mice serum **(A)** and tissues **(B)**. Histopathological examination of the liver in control mice (I) and mice treated with 125 mg/kg (II), 250 mg/kg (III) and 500 mg/kg (IV) corynoline for 24 h **(C)**. Concentration of corynoline in plasma and six tissues at 0.5, 2, 4, and 8 h after an intragastric administration of corynoline (250 mg/kg) in healthy mice **(D)**. Data are *mean* ±*SD*.

Compared with the control group, ALT and AST in serum and livers of the mice were not significantly different (*P* > 0.05) in drug treatment groups, at 24 h after oral administration of 125, 250, and 500 mg/kg CRL (see Figures 6A,B). Moreover, the histopathological examination showed that no pathological changes were found in the liver of the mice in each experimental group (see Figure 6C). The results indicated that CRL had no obvious hepatotoxicity and did not induce acute liver injuries in the experimental dosage used in this study. However, compared with control group, abnormal behaviors of mice in drug treatment groups were observed, including lying on the bedding and shivering at about 10 min after administration (more frequently at the beginning and occasionally later). Besides, the body temperature of most mice in drug treatment groups was lower than normal. The phenomena were more pronounced with the dosage increasing. Some mice gradually returned to normal at 3 h after administration in low dosage group. Few mice returned to normal in middle and high dosage groups at 10 h after administration and one mouse in middle dosage group and two mice in high dosage group were dead.

### Tissue Distribution of CRL in Mice

The LC-MS/MS methods for quantifying CRL in plasma and tissues were evaluated, and were successfully applied to the tissue distribution study of CRL in mice. Concentration of CRL in plasma and six tissues at 0.5, 2, 4, and 8 h after an intragastric administration of CRL (250 mg/kg) in mice were shown in Figure 6D. The highest concentration levels of CRL detected in each of the tissues and the successive order from high to low of them were liver (92.91 ± 65.11) μg/g, kidney (68.73 ± 46.89) μg/g, heart (48.76 ± 46.89) μg/g, brain (40.53 ± 20.50) μg/g, lung (27.27 ± 22.17) μg/g, and spleen (25.22 ± 18.67) μg/g. The time to reach the maximum concentration of CRL was at 2 h post dosing. The concentration of CRL in plasma was (18.35 ± 14.16) μg/mL at 2 h post dosing. It is worth noting that CRL could traverse the blood–brain barrier, and have relative high concentration in brain. In short, the main target organ of CRL was liver, then kidney, heart, and brain.

### Metabolic Activation of CRL in Rats

#### Characterization of CRL Metabolites

**FIGURE 7:**
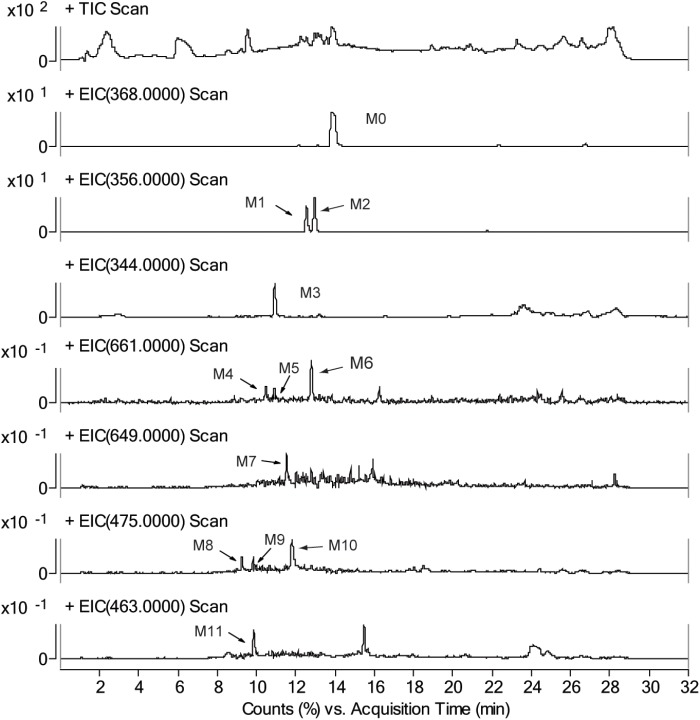
Total ion chromatograms and extracted ion [M+H]^+^ chromatograms of corynoline (M0), metabolites (M1, M2, and M3), their GSH conjugates (M4, M5, M6, and M7) and their cysteine (Cys) conjugates (M8, M9, M10, and M11) in mouse liver samples.

**FIGURE 8:**
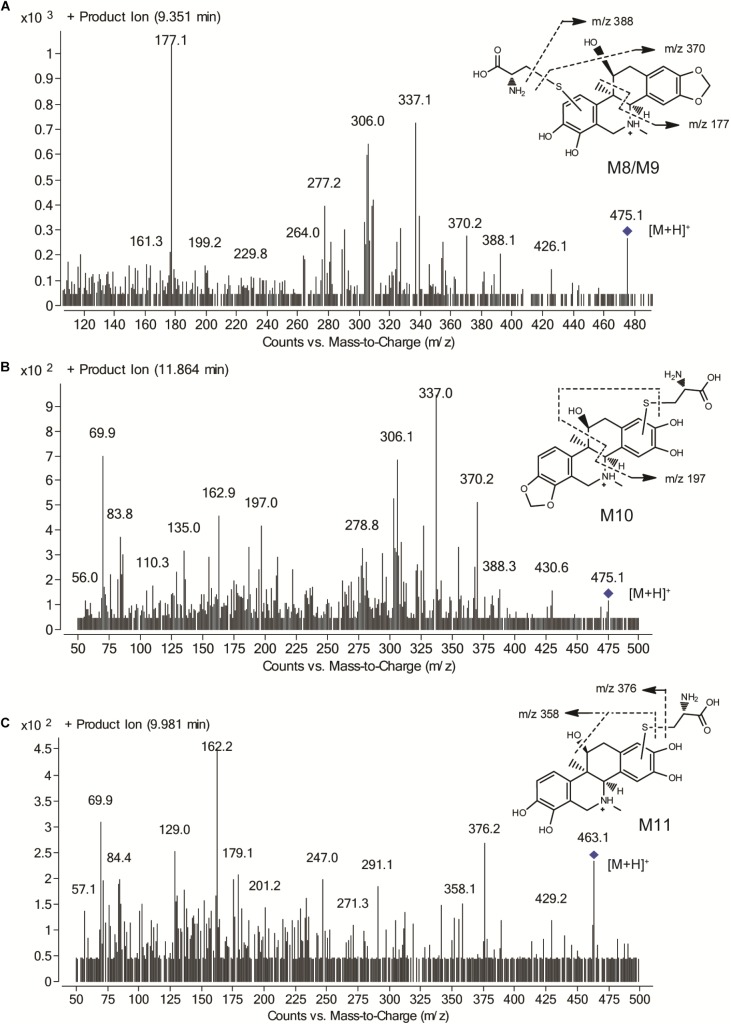
Mass spectrum in product ion mode of corynoline reactive metabolite cysteine adducts in mouse liver samples, including M8/M9 **(A)**, M10 **(B)** and M11 **(C)**.

Identifying the metabolites of CRL in liver samples of mice was performed by using LC-MS/MS. M1-M7 were found in liver just the same as those *in vitro*. Besides, four new metabolite-cysteine conjugates were identified in liver samples, named as M8–M11 (Figure 7), which were corresponding to M4–M7 with the GSH displayed by cysteine. Mass spectrum in product ion mode of CRL reactive metabolite cysteine adducts (labeled as M8–M11) in mouse liver samples were shown in Figure 8. The [M+H]^+^ of M8–M10 (m/z 475), M11 (m/z 463) were more 119 Da than those of M1/M2 (m/z 356), M3 (m/z 344) respectively, and we speculated that M8–M11 were the conjugates of CRL metabolites and cysteine. m/z 388 and m/z 370 were fragment ions of M8–M10, and the m/z 388 was formed by breaking the S-C chemical bond in cysteine, and m/z 370 was formed by losing one H_2_O from B ring of m/z 388. Fragment ion m/z 177 was found in M8 and M9, and m/z 197 was found in M10, which indicated that the binding site of cysteine with CRL metabolites in M8-M10 was corresponding to that of GSH with CRL metabolites in M4–M6. Fragment ion m/z 376 of M11 was formed by breaking the S-C chemical bond in cysteine, and m/z 358 was formed by losing one H_2_O from B ring of m/z 376. This was further evidence that M11 was the conjugate of cysteine and *ortho*-benzoquinone intermediate of CRL.

#### Semi-quantification of CRL Metabolites in Mouse Liver

The relative content of CRL metabolite-GSH/Cys conjugates in the liver samples obtained in tissue distribution study was determined by using LC-MS/MS with multiple reaction monitoring model. The content of conjugates was expressed using the peak area of analytes (Supplementary Table 5). M6 and M10 were found in relatively high levels in the mice liver.

## Discussion

In this study, the metabolites and *ortho*-benzoquinone intermediate-GSH conjugates of CRL were identified by LC-Q-TOF/MS and LC-MS/MS. Three metabolites (M1, M2, and M3) of CRL were identified in the incubation system with rat or human microsomes, which was consistent with the results reported by Mao et al. (2015). Besides, reactive electrophilic metabolites are generally short lived and not easy to detect. The formation of reactive metabolites can be inferred from conjugates derived from reaction with endogenous nucleophiles (Schmidt et al., 2013; Huang et al., 2015). Reactive metabolites were found using GSH as the trapping agent and four GSH conjugates (M4–M7) were detected by LC-Q-TOF/MS. It could be inferred M4–M7 were derived from M1–M3 conjugated with one GSH molecular, respectively, according to the information of the mass spectrum. Besides, the GSH conjugates (M4–M7) were further confirmed by using the neural loss mode (129 Da) with positive ionization and precursor ion mode (272 Da) with negative ionization of LC-MS/MS.

Furthermore, the CYP450 isoforms involved in CRL metabolic activation were identified by detecting the *ortho*-benzoquinone intermediate-GSH conjugates. The results indicated that CYP3A4, CYP2C19, CYP2C9, and CYP2D6 mediated the formation of CRL reactive metabolites. Therefore, the metabolism of CRL may be affected when it was combined with the inhibitor or inductor of these CYP450 enzymes, and the drug interaction may occur.

Many studies have indicated that drugs or xenobiotics which produced metabolic activation possibly result in idiosyncratic drug toxicity, in particular, liver toxicity (Carvalho et al., 2004; Erve et al., 2013; Amaya et al., 2018). Hence, hepatotoxicity evaluation, tissue distribution and metabolism of CRL in mice were conducted in our study. The tissue distribution study of CRL in mice indicated that liver was the main target organ of CRL. Moreover, *ortho*-benzoquinone intermediate-GSH and -cysteine conjugates were both detected in mice liver samples, which suggested reactive metabolites of CRL could covalently bind to the cysteine residues *in vivo* besides GSH. That may disrupt normal function of the organ or tissue and result in the related toxicity. Then, the toxicity evaluation results showed that CRL could not cause acute liver injury with the experimental dosage. It is worth noting, however, that some mice exhibit abnormal behaviors and three of them die. We surmise that toxicity effect of CRL on other organs may have occurred, and more attention should be paid on the traditional Chinese medicine contained CRL in clinic. It is not clear whether it is related to that CRL could through the blood–brain barrier, and if the bioactivation of CRL have associated with the induce toxicity. Furthermore, the relative content of CRL metabolite-GSH/Cys conjugates in the liver samples were determined by using LC-MS/MS, and the results showed that the levels of M6 and M10 were the highest in mice liver. And in addition to these we also found that CYP3A was the major contributor for the formation of M6, with the contribution rate of 73.5%. Therefore, the metabolism of CRL mediated by CYP3A was also a noteworthy topic.

## Conclusion

In summary, this study has comprehensively investigated the metabolism of CRL and evaluated its hepatotoxicity in mice for the first time. Our findings showed that metabolic activation of CRL occurred in mice, and the main target organ of CRL was liver, then kidney, heart, and brain. CRL could traverse the blood–brain barrier and toxicity effect of CRL in mice was observed, though the hepatotoxicity was not detected in mice under experimental dosages. Therefore, more attention should be paid on the traditional Chinese medicine contained CRL in clinic. Further studies are needed to be conducted for better understanding the toxicity of CRL and the relationship between the toxicity and its metabolism.

## Author Contributions

RL, XT, and LD designed the experiments. RL, FZ, HH, and JW conducted the experiments, analyzed the data, and wrote the paper. All authors contributed to the editing of the paper and to scientific discussions.

## Conflict of Interest Statement

The authors declare that the research was conducted in the absence of any commercial or financial relationships that could be construed as a potential conflict of interest.
