# Supplementary material for: Metabolism and Bioactivation of Corynoline With Characterization of the Glutathione/Cysteine Conjugate and Evaluation of Its Hepatotoxicity in Mice
Source: Front Pharmacol. 2018 Nov 6;9:1264. doi: 10.3389/fphar.2018.01264 (PMC6232370; doi:10.3389/fphar.2018.01264)
Supplement: Supplementary file 1 [file Table_1.docx]

Supplementary Material

Metabolism and bioactivation of corynoline with characterization of the glutathione/cysteine conjugate and evaluation of its hepatotoxicity in mice

Ruijuan Liu, Fang Zhou, He He, Xin Tian*, Li Ding*

*** Correspondence:** Xin Tian: tianx@zzu.edu.cn, Li Ding: dinglidl@hotmail.com.

# Supplementary Figures and Tables

## Supplementary Figures


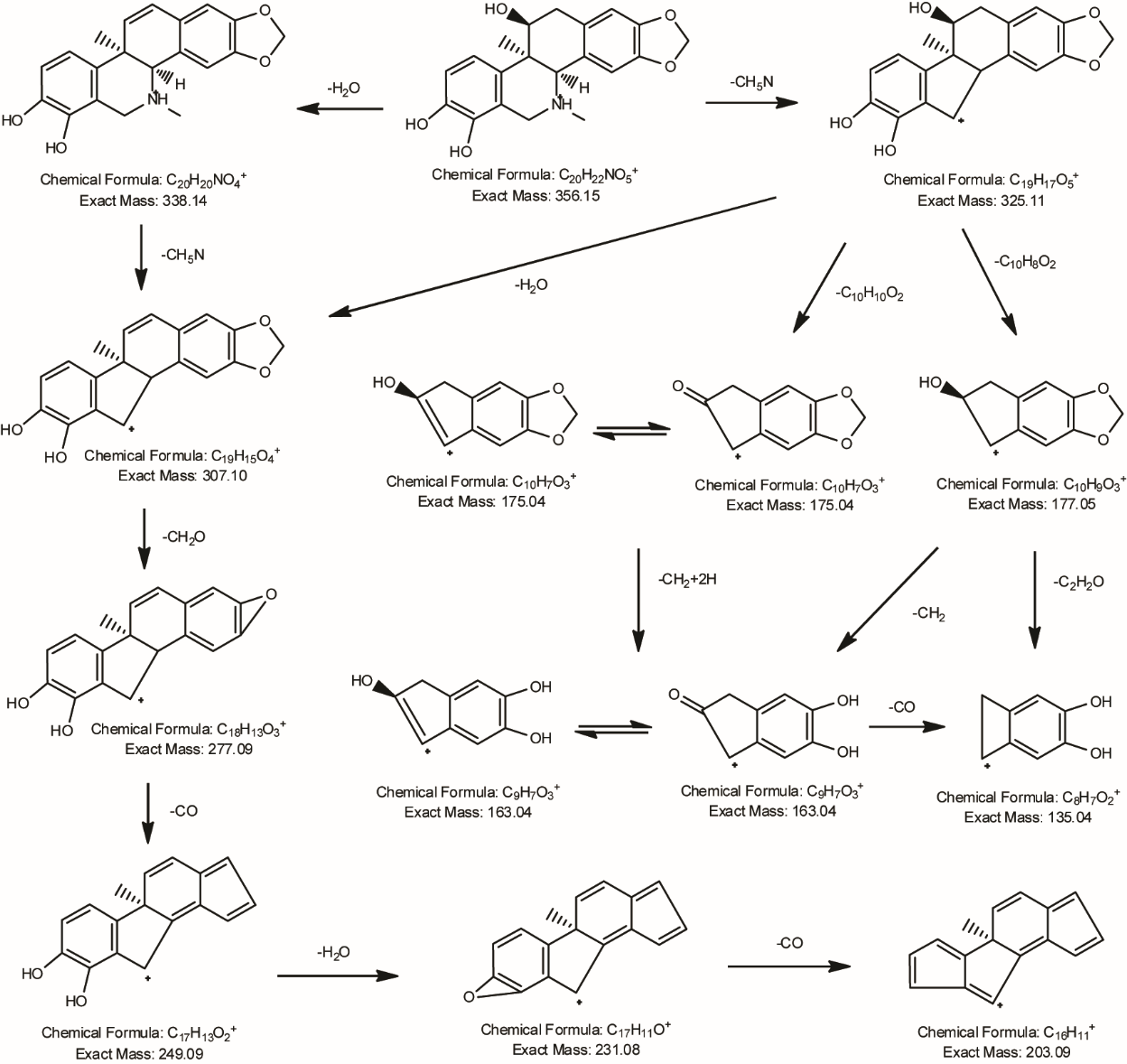


**Supplementary Figure 1** Proposed structure and fragmentation pathways of M1


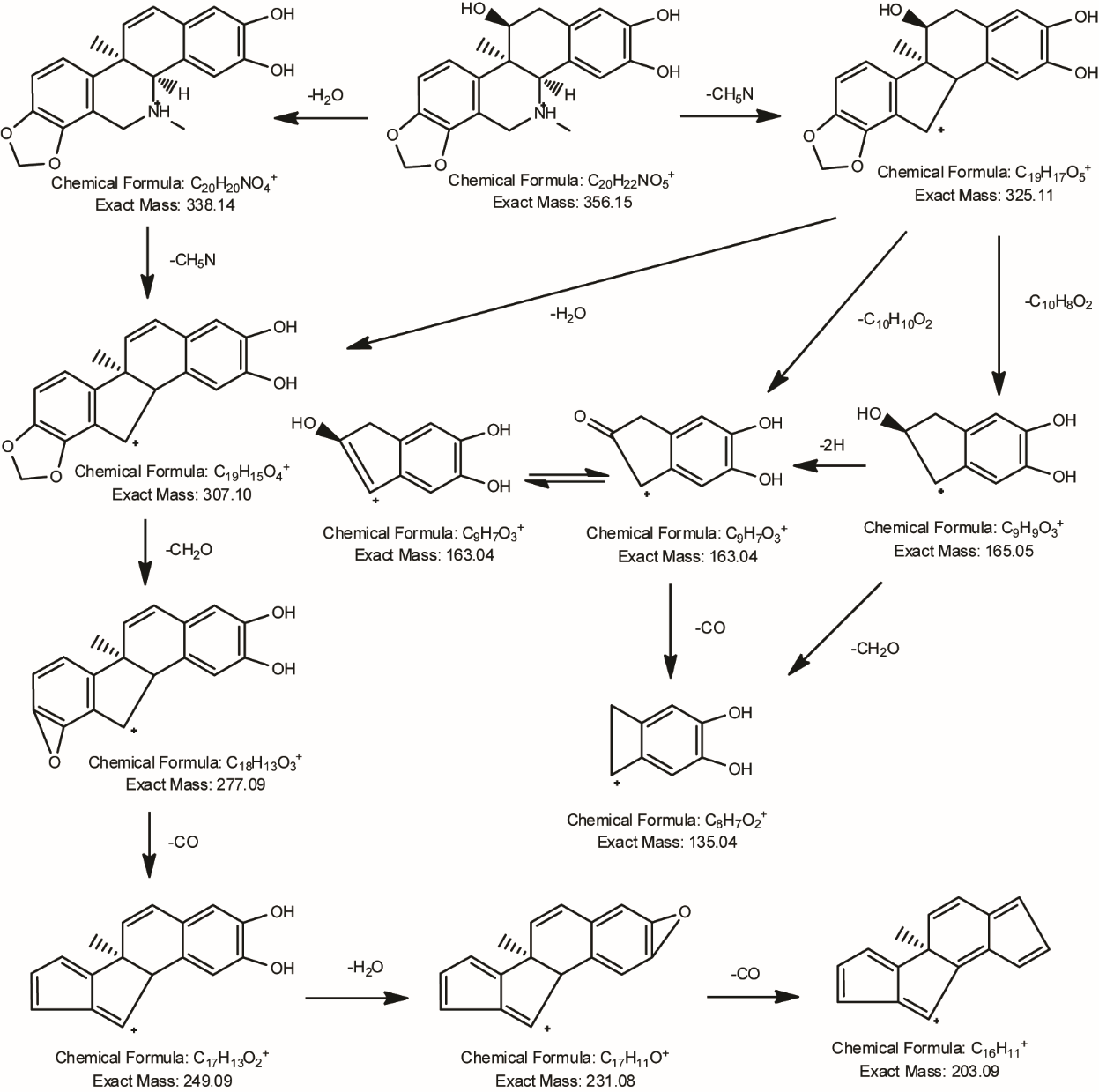


**Supplementary Figure 2** Proposed structure and fragmentation pathways of M2


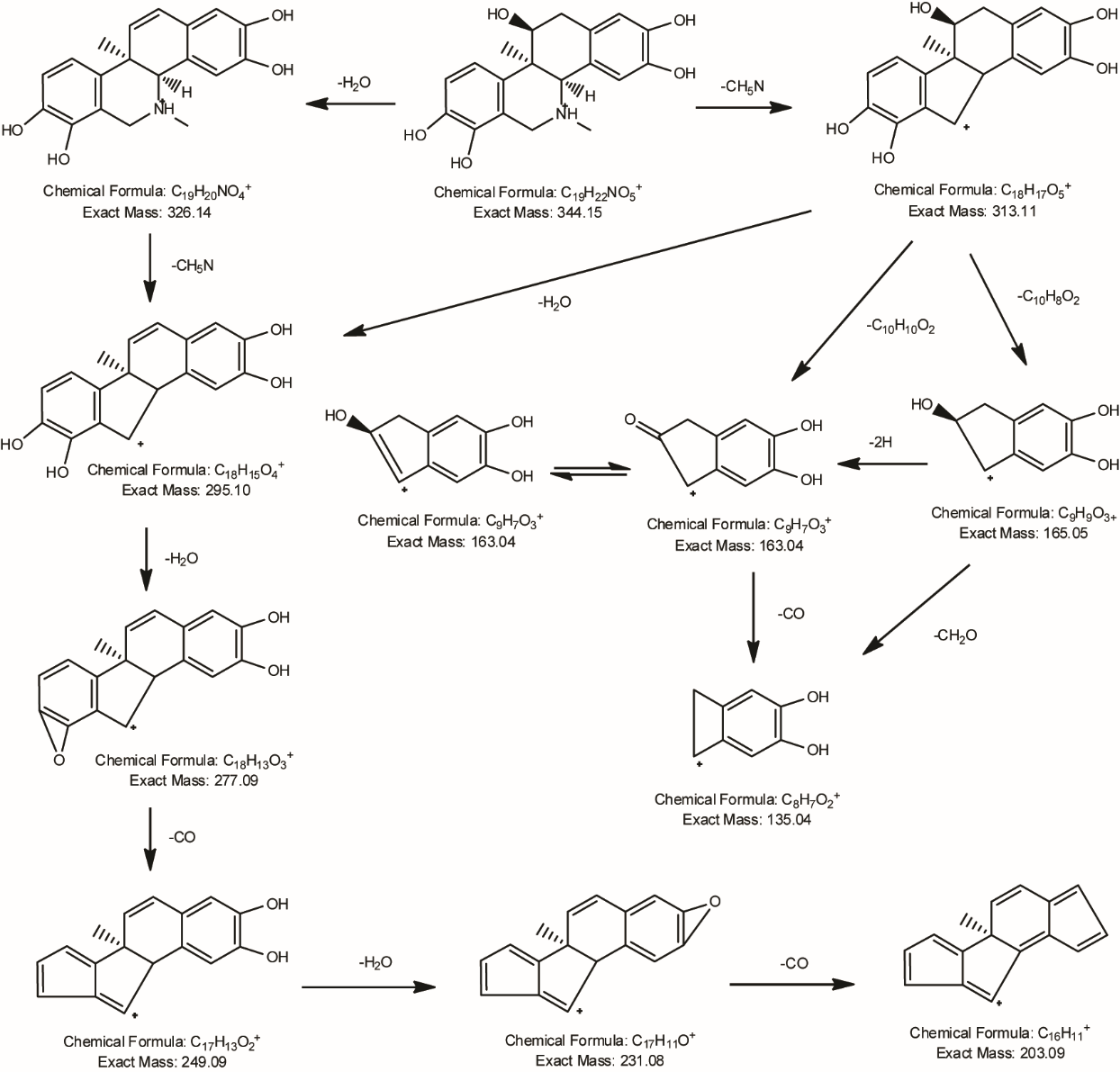


**Supplementary Figure 3** Proposed structure and fragmentation pathways of M3


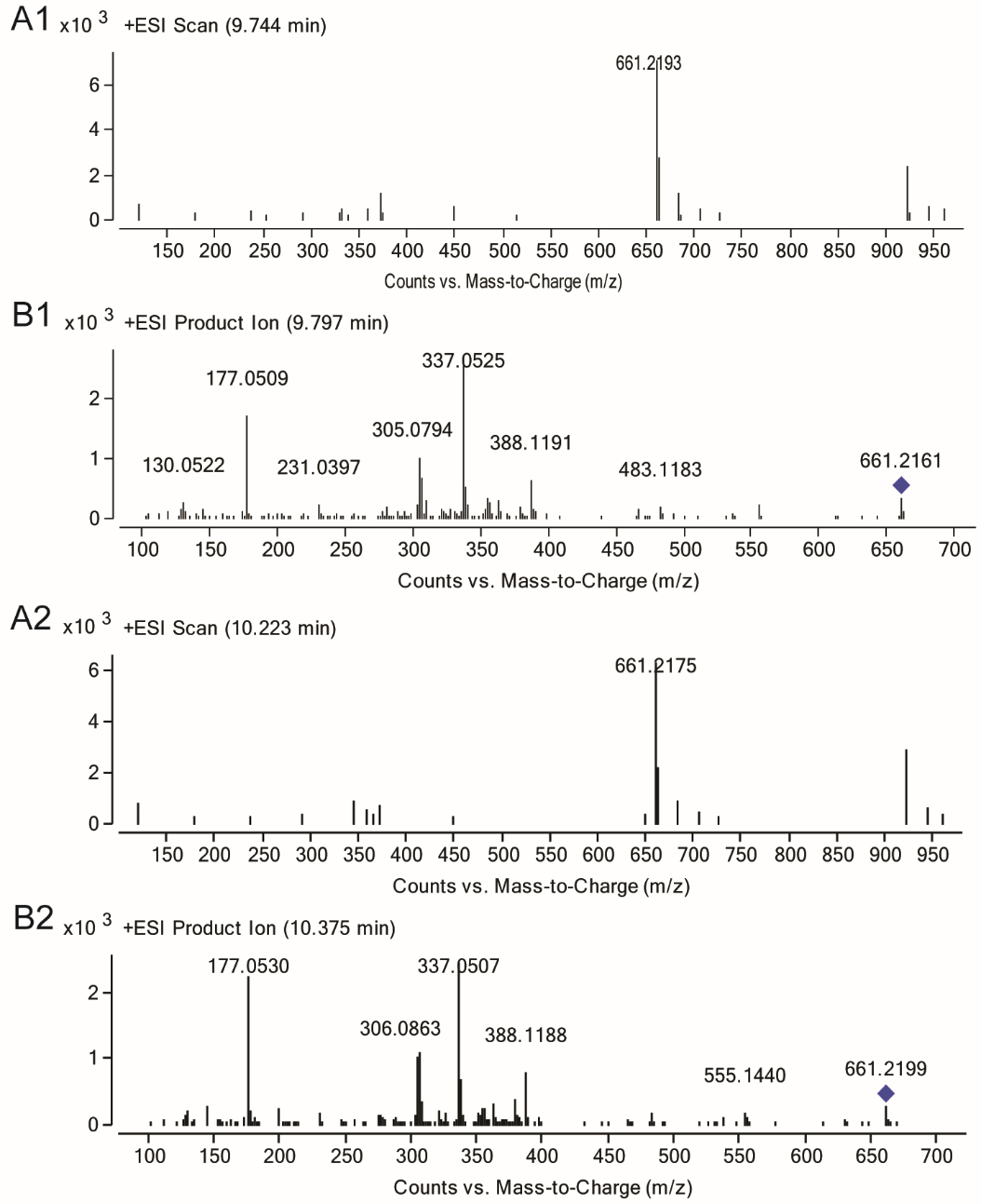


**Supplementary Figure 4** The mass spectra of M4, M5 in full scan (A1 and A2) and product ion scan (B1 and B2) with the precursor ion of [M+H]^+^


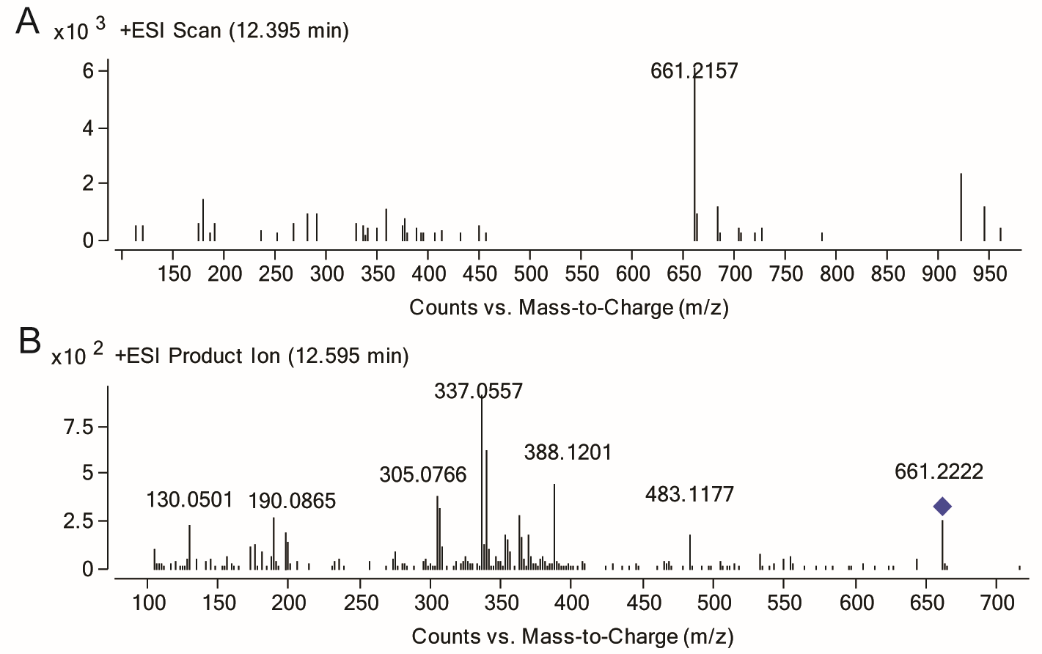


**Supplementary Figure 5** The mass spectra of M6 in in full scan (A) and product ion scan (B) with the precursor ion of [M+H]^+^


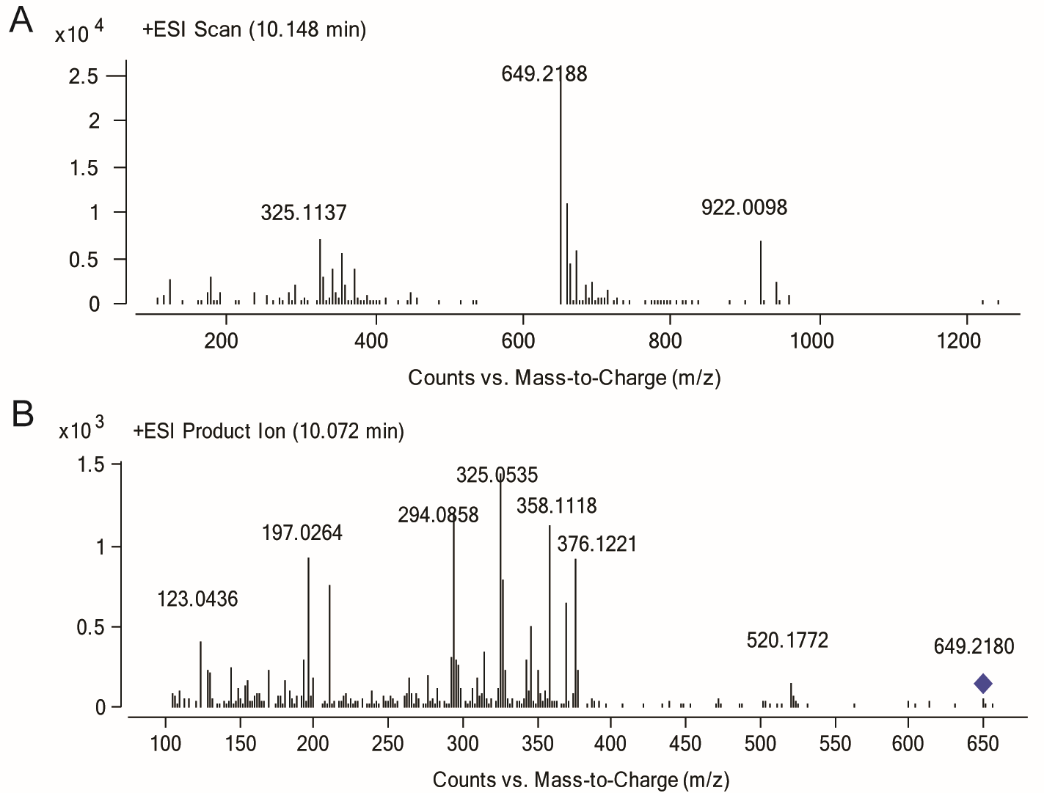


**Supplementary Figure 6** The mass spectra of M7 in full scan (A) and product ion scan (B) with the precursor ion of [M+H]^+^

**
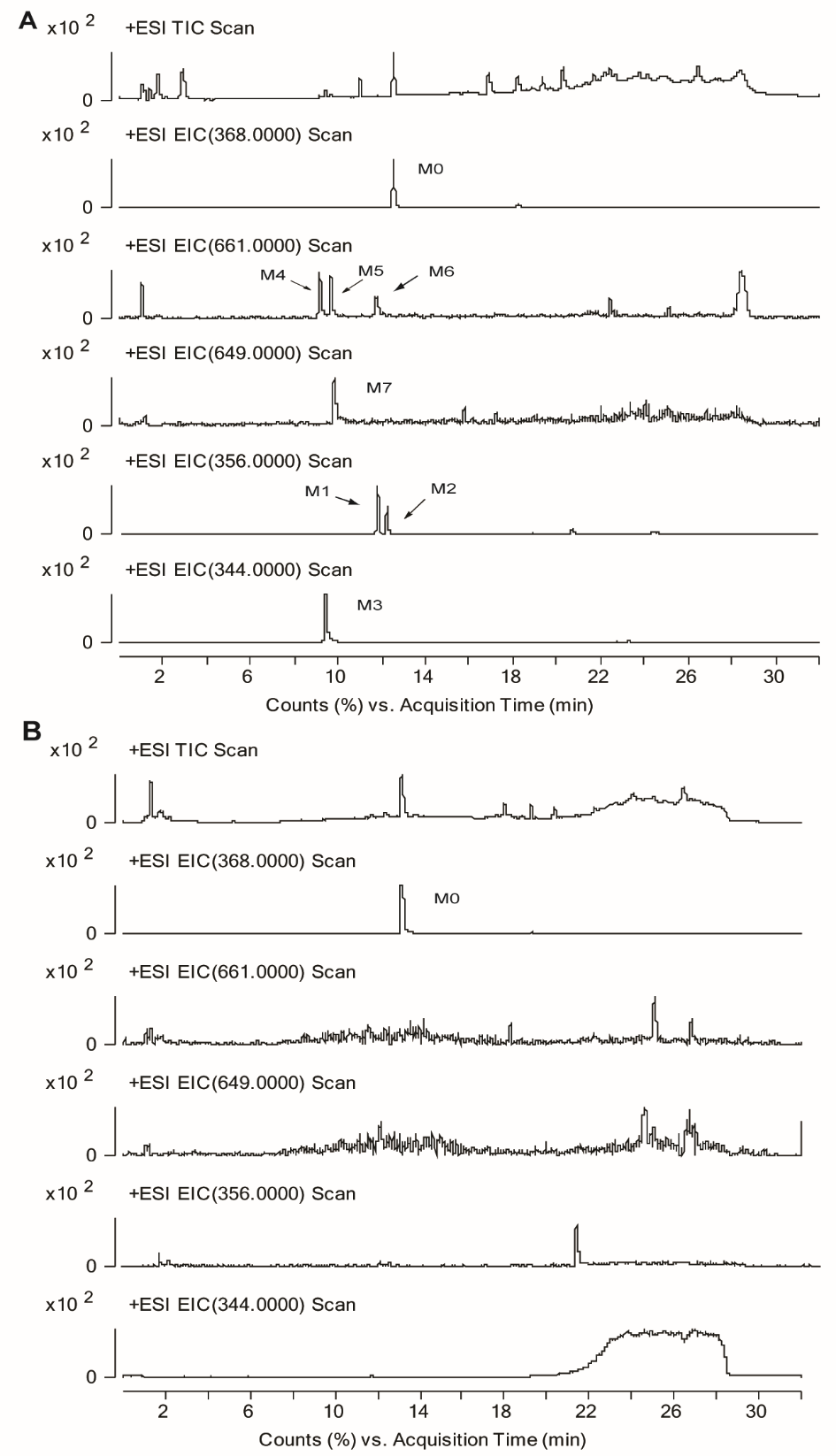
**

**Supplementary Figure 7** Total ion chromatograms and extracted ion [M+H]^+^ chromatograms of corynoline (M0), metabolites (M1, M2, M3) and theirs GSH conjugates (M4, M5, M6, M7) in human liver microsome incubations. (A) with NADPH and GSH, (B) without NADPH.

## Supplementary Tables

**Supplementary Table 1** Information of the [M+H]^+^ of M1 and its product ions in mass spectrum of product ion mode

| Observed *m/z* | Formula | Calculated *m/z* | Diff (ppm) | Loss Formula |
| --- | --- | --- | --- | --- |
| 356.1479 | C_20_H_21_NO_5_ | 356.1492 | 3.80 | - |
| 338.1372 | C_20_H_19_NO_4_ | 338.1387 | 4.40 | H_2_O |
| 325.1047 | C_19_H_17_O_5_ | 325.1071 | 7.20 | CH_4_N |
| 307.0952 | C_19_H_15_O_4_ | 307.0965 | 3.96 | CH_6_NO |
| 277.0848 | C_18_H_13_O_3_ | 277.0859 | 3.97 | C_2_H_8_NO_2_ |
| 249.0896 | C_17_H_13_O_2_ | 249.091 | 5.76 | C_3_H_8_NO_3_ |
| 231.0797 | C_17_H_11_O | 231.0804 | 3.00 | C_3_H_10_NO_4_ |
| 177.0532 | C_10_H_9_O_3_ | 177.0546 | 8.15 | C_10_H_12_NO_2_ |
| 163.0400 | C_9_H_7_O_3_ | 163.039 | 6.50 | C_11_H_14_NO_2_ |
| 135.0434 | C_8_H_7_O_2_ | 135.0441 | 4.95 | C_12_H_14_NO_3_ |
| 123.0427 | C_7_H_7_O_2_ | 123.0441 | 11.02 | C_13_H_14_NO_3_ |

**Supplementary Table 2** Information of the [M+H]^+^ of M2 and its product ions in mass spectrum of product ion mode

| Observed *m/z* | Formula | Calculated *m/z* | Diff (ppm) | Loss Formula |
| --- | --- | --- | --- | --- |
| 356.1490 | C_20_H_21_NO_5_ | 356.1492 | 0.70 | - |
| 338.1381 | C_20_H_19_NO_4_ | 338.1387 | 1.73 | H_2_O |
| 325.1052 | C_19_H_17_O_5_ | 325.1071 | 5.69 | CH_4_N |
| 307.0961 | C_19_H_15_O_4_ | 307.0965 | 1.25 | CH_6_NO |
| 277.0847 | C_18_H_13_O_3_ | 277.0859 | 4.41 | C_2_H_8_NO_2_ |
| 249.0892 | C_17_H_13_O_2_ | 249.091 | 7.25 | C_3_H_8_NO_3_ |
| 231.0798 | C_17_H_11_O | 231.0804 | 2.78 | C_3_H_10_NO_4_ |
| 165.0539 | C_9_H_7_O_3_ | 165.0546 | 4.37 | C_11_H_14_NO_2_ |
| 135.0437 | C_8_H_7_O_2_ | 135.0441 | 2.69 | C_12_H_14_NO_3_ |
| 123.0433 | C_7_H_7_O_2_ | 123.0441 | 6.14 | C_13_H_14_NO_3_ |

**Supplementary Table 3** Information of the [M+H]^+^ of M3 and its product ions in mass spectrum of product ion mode

| Observed *m/z* | Formula | Calculated *m/z* | Diff (ppm) | Loss Formula |
| --- | --- | --- | --- | --- |
| 344.1488 | C_19_H_21_NO_5_ | 344.1492 | 1.31 | - |
| 326.1379 | C_19_H_19_NO_4_ | 326.1387 | 2.41 | H_2_O |
| 313.1102 | C_18_H_17_O_5_ | 313.1071 | -9.28 | CH_4_N |
| 295.0960 | C_18_H_15_O_4_ | 295.0965 | 1.40 | CH_6_NO |
| 277.0854 | C_18_H_13_O_3_ | 277.0859 | 2.04 | CH_8_NO_2_ |
| 249.0881 | C_17_H_13_O_2_ | 249.091 | 11.67 | C_2_H_8_NO_3_ |
| 231.0789 | C_17_H_11_O | 231.0804 | 6.67 | C_2_H_10_NO_4_ |
| 165.0560 | C_9_H_7_O_3_ | 165.0546 | -8.36 | C_10_H_14_NO_2_ |
| 123.0434 | C_7_H_7_O_2_ | 123.0441 | 5.33 | C_12_H_14_NO_3_ |

**Supplementary Table 4** Contributions of recombinant CYP450 enzymes for reactive metabolites formation of corynoline

| CYP450 | Contribution rate (%) | | | |
| --- | --- | --- | --- | --- |
|  | M4 | M5 | M6 | M7 |
| 1A2 | 0.6 | 0.0 | 3.6 | 0.0 |
| 2B6 | 0.0 | 0.0 | 0.4 | 0.0 |
| 2C8 | 3.1 | 2.1 | 2.4 | 0.0 |
| 2C9 | 20.1 | 30.4 | 10.8 | 0.0 |
| 2C19 | 23.6 | 28.5 | 5.7 | 32.7 |
| 2D6 | 13.0 | 3.7 | 3.1 | 26.0 |
| 3A4 | 39.4 | 35.1 | 73.5 | 40.2 |
| 3A5 | 0.4 | 0.2 | 0.5 | 0.2 |
| 2E1 | 0.0 | 0.0 | 0.0 | 0.0 |

**Supplementary Table 5** Relative quantification of corynoline and its reactive metabolites in mouse liver samples

| Time (h) | Peak aera of analytes | | | | | |
| --- | --- | --- | --- | --- | --- | --- |
|  | M4 | M5 | M6 | M7 | M10 | M11 |
| 0.5 | 8520 | 2784 | 45665 | 266 | 44725 | 412 |
| 2 | 6751 | 2569 | 35143 | 230 | 38737 | 570 |
| 4 | 7935 | 2765 | 26779 | 165 | 48061 | 446 |
| 8 | 7235 | 2302 | 23418 | 160 | 46688 | 463 |
